# Can We Expect Results-Based Financing to Improve Quality of Care?

**DOI:** 10.9745/GHSP-D-17-00069

**Published:** 2017-03-15

## Abstract

Performance-based incentives as currently employed appear poorly adapted for improving quality of clinical processes. They mainly measure structural items that, while easier to measure, are remote from actual clinical quality, and they could even perversely lead to heightened attention to those factors at the expense of clinical quality.

See related article by Gergen.

Results-based financing (RBF) schemes in health care are premised on the notion that “paying for results” rather than for inputs is more likely to improve performance. But getting from that attractive hypothesis to program efforts that improve quality of care and outcomes at population scale—in the real world—is far from simple.

The article by Gergen^1^ in this issue of GHSP offers an overview of a set of RBF schemes, mainly funded under the World Bank's Health Results Innovation Trust Fund (HRITF). Under this funding, performance-based incentives are paid to health workers and health facilities, with the intention of improving quality of health services for women and children. As of late 2016, this major funding program, supported primarily by the governments of Norway and the United Kingdom, has provided close to US$400 million in grants and more than US$2 billion in associated loans to RBF programs in 29 countries.^2^

## STRUCTURE VS. PROCESS VS. OUTCOME

Incentives, as used in these schemes, get health worker attention but may not direct attention where it's most needed. As the Gergen paper acknowledges, in these financing schemes quality is operationalized largely in terms of *structure*, rather than *processes* or *outcomes* of care (using the language of the Donabedian model^3^). Among the 54 most common indicators tracked by the checklists used in the schemes Gergen et al. review, the large majority relate to what they describe as “structural quality.” The 4 most frequently used are:

- Latrines/toilets present and in good working order
- Performance or activity reports submitted on time
- Financial and accounting documents available and well kept
- Fencing around the building existent and well kept

Important though such indicators may be, it's not at all clear that in the aggregate they constitute an adequate account of the causal factors that can be expected to drive improved *clinical processes* and *outcomes*. Indeed, in the Donabedian model, it is the additive effect of inputs and care processes that yield improved health outcomes. Using terms from the Institute of Medicine's conceptualization of health care quality, the focus needs to be on ensuring that *actual clinical care* provided to every client is safe, effective, patient-centered, timely, efficient, and equitable.^4^

To be sure, among the RBF performance indicators presented by Gergen et al., a few attempt to get at what care is actually provided and there is frequent discussion among RBF proponents about how to incorporate more quality of care process measures into financial incentive schemes in low-resource settings.

But, as described by Gergen et al., the data used for verification in the schemes they review are largely limited to information abstracted from routine registers and patient records; very little is based on direct observation or client exit interviews. In most instances, review of routine documents as described in the Gergen article will be inadequate to assess clinical quality or appropriateness of care (e.g., “Integrated Management of Childhood Illness [IMCI] protocol is applied correctly”).

## WHAT'S IMPORTANT MAY NOT BE READILY MEASURABLE; WHAT'S MEASURABLE MAY NOT BE IMPORTANT

We can assume that those developing performance measures for these funding schemes would have very much liked to have more and better measures of actual quality of care. Unlike tracking of service volume, however, clinical quality of care tends to be difficult to measure. This challenge is particularly acute in low-resource settings where primary patient data are often absent (e.g., lack of standardized patient records, stock-outs of registers) and routine health information systems often include few quality of care clinical process and health outcome measures. Concomitantly trying to measure quality of care while building health information systems capable of measuring quality is a common challenge faced by programs implementing quality improvement and financial incentive schemes alike.

But if we are seeking to incentivize based on measures of performance, unless we use measures that closely approximate what we're most interested in influencing, we risk misdirecting effort toward factors that are less likely to contribute to improvements on meaningful endpoints and away from potentially more important unmeasured factors.

Unlike tracking of service volume, clinical quality of care tends to be difficult to measure.

## THE FULL SET OF CONDITIONS REQUIRED FOR IMPROVED OUTCOMES

Moving back a step on the causal chain from the process of clinical care, we have a set of conditions that need to be met if appropriate, quality care is to be provided for every client every time. This has been referred to as “implementation strength.”^5^ One simple way to think about the immediate proximal set of such factors is to remember the acronym ACME; for a specific service to be delivered such that it produces its desired benefit, systems need to be functional such that health workers are:

- **A**vailable to those needing the service
- **C**apable, i.e., have the knowledge and skills required for that particular service
- **M**otivated to provide the service
- **E**nabled, i.e., have the necessary infrastructure, equipment, drugs, and other supplies

In order to provide quality care, health workers need to be available, capable, motivated, and enabled.

Not all of this will be easily measurable, but any scheme aiming to bring about improved individual- and population-level health outcomes must seriously come to grips with the conditions that need to be satisfied to achieve such improvements.

## PERFORMANCE: THE REAL THING OR ONLY THE APPEARANCE?

For RBF schemes to be effective, those designing and delivering them need to be clear-eyed about what behaviors they are actually incentivizing. Like measurement of quality, rigorous, independent verification can be difficult and costly. But without such verification, it is not possible to know whether these schemes are, in fact, contributing to improved quality of care. In the absence of clear evidence for the effect of financial incentives on improved quality of care and health outcomes in low-resource settings,^6^ it is more important than ever to pursue rigorous assessments of the effect and costs of implementing such schemes.

Incentive schemes risk rewarding the mere appearance of improved performance. It is not clear that implementation of these schemes has been accompanied by adequate safeguards against complicity, for example, between those in the health facility being incentivized and their peer-reviewers.

## WHAT'S MISSING FROM THIS PICTURE?

On finishing the Gergen article, the reader may be left wondering:

- Why should one believe that incentivizing primarily structural factors will necessarily lead to improvement in clinical processes or outcomes?
- Even if there were an impact on quality or outcomes, to what extent would it prove feasible to replicate and sustain such results at scale?
- Implemented at scale, strong data validation mechanisms (like the rigorous impact evaluations done when these schemes were first piloted^7^) would be difficult to sustain. In the absence of such strong validation, won't there be a tendency to try to game the system since maintaining the appearance of good performance will often be easier than producing the real thing?
- For improving quality, what is more likely to be effective: (1) data use within the health facility for directing problem solving, or (2) documenting and reporting data to submit elsewhere, serving as a basis for an incentive calculation? Or both? How compatible are these 2 approaches to improving performance?
- How consistent are performance-based incentives, as used in these schemes, with known best practices in the quality improvement field?^8^

In fairness, RBF schemes are typically not just about quality improvement; they are concerned with program performance more broadly defined. But surely we are kidding ourselves if we think that measuring and incentivizing performance operationalized mainly in terms of “structural” factors will take us very far toward improved clinical care and health outcomes. –*Global Health: Science and Practice*

## Notes

**First Published Online:** 2017 Mar 15
